# Extracellular enzymes producing yeasts study: cost-effective production of α-amylase by a newly isolated thermophilic yeast *Geotrichum candidum* PO27

**DOI:** 10.3934/microbiol.2024006

**Published:** 2024-01-29

**Authors:** Ibtissem Chaib, Scheherazed Dakhmouche-Djekrif, Leila Bennamoun, Tahar Nouadri

**Affiliations:** 1 Laboratory of Microbiological Engineering and Applications, Department of Biochemistry and Molecular and Cellular Biology, Faculty of Natural and Life Sciences, Frères Mentouri University Constantine 1, Constantine 25017, Algeria; 2 Department of Natural Sciences, Teachers Training School El Katiba ‎Assia Djebar, University town Ali Mendjeli, Constantine 25000, Algeria

**Keywords:** *Geotrichum candidum* PO27, α-amylase, Olive pomace, SSF, SmF, OFAT

## Abstract

Enzymes are biocatalysts mainly used for their industrial potential in various applications. The present study aims to understand the enzyme production for biotechnological interest from a local yeast strain. From 100 isolates obtained from various biotopes, 78 strains were selected for their enzymatic heritage. Screening of α-amylase, lipase/esterase, and cellulase activities by rapid plate detection methods was carried out and the PO27 yeast was selected for its high capacity to produce α-amylase. In addition, this yeast strain exhibited good lipolytic and esterolytic activities, as well as low cellulase activity. A sequence analysis of the D1/D2 region of the 26S ribosomal RNA (26S rRNA) and a study of morphological characteristics identified the PO27 strain as *Geotrichum candidum*. The production of α-amylase has been studied in solid medium fermentation using various natural substrates without any supplementation such as olive pomace, potato peels, leftover bread, and mastic cake. *G. candidum* PO27 showed an improved production of α-amylase with olive pomace, thus reaching approximately 180.71 U/g. To evaluate the ability of this isolate to produce α-amylase in submerged fermentation, multiple concentrations of olive pomace substrate were tested. The best activity of submerged fermentation was statistically compared to the solid-state fermentation result in order to select the appropriate fermentation type. A high significant difference was found to rank the 6% olive pomace medium as the best substrate concentration with 34.395 U/mL of α-amylase activity. This work showed that the new isolate *Geotrichum candidum* PO27 has a better potential to produce α-amylase at a low cost in solid-state fermentation compared to submerged fermentation. Optimization conditions for PO27 α-amylase production through solid-state fermentation were achieved ‎ using a one factor at a time (OFAT) approach. The findings revealed that a high temperature (60 °C), an acidic pH, malt extract, and soluble starch were the highly significant medium components for enhancing α-‎amylase production. The use of olive pomace waste by *Geotrichum candidum* PO27 is expected to be effective in producing an industrially useful α-amylase.

## 1. Introduction

The demand for industrial enzymes is continuously growing worldwide, in which the net worth of approximately $0.31 billion in 1960 was raised to $4.9 billion in 2015, to $6 billion in 2020, and is expected to reach more than $9 billion USD by 2027. This has led to significant success for many enzyme companies [1]–[3]. These enzymes are widely secreted by plants, animals, and microorganisms. However, microbial enzymes have received more attention due to their easy availability, rapid growth, and an active and stable nature, as well as their high yield on inexpensive media and in a shorter time and a straightforward production by recombinant DNA technology using microbes as the host cells [4],[5]. Most microorganisms are unable to produce enzymes under challenging conditions that induce microorganism toxicity. However, some microorganisms with thermostable enzyme systems have undergone various adaptations that allow them to grow and produce enzymes under harsh conditions, mainly temperature and pH, to be commercially attractive [6],[7].

Recently, several studies have been initiated to isolate many potential enzyme-producing microorganisms, including bacteria, fungi, and yeast strains from several sources. They can be isolated from soil, water [8],[9], hot springs [10], fruits, wastes from the palm oil plant [11], marine environments [12], and oil mill effluent [13],[14]. In recent years, yeasts have emerged as a major source of enzymes, in addition to their high lipid production capacity, short fermentation cycles, independence of climatic growth, low pH value, and growth capabilities on a large variety of substrates [15]. It has been found that yeasts producing enzymes such as α-amylase, lipase, cellulase, and esterase are found everywhere in nature. These enzymes are applied in several industrial processes, such as textiles, leather, paper and pulp, research and development, pharmaceutical, agriculture, detergent, waste, biorefineries, photography, and food industries [3]; with these attractive remarkable characteristics, they are particularly considered as an ideal biocatalyst.

The production of enzymes such as α-amylase using synthetic media is very expensive and an uneconomical, low-cost medium is required to meet the demand of industries. For these reasons, researchers have investigated agro wastes such as potato peels, bread waste, and oil cakes [16]–[18] as an alternative substrate to replace the high-cost media employed through submerged fermentation (SmF) and solid-state fermentation (SSF). In addition, it helped to reduce the environmental pollution caused by their disposal in the fields, which could create a serious environmental problem due to the phytotoxic nature of their compounds. To reduce the cost of enzymes, it would be interesting to isolate local enzyme-producing yeast strains with new characteristics. The selected *Geotrichum candidum* PO27 can use various wastes as an alternative carbon source for α-amylase production under SSF and SmF, which is an economic advantage since the application of these enzymes is increasing in several industries. However, a few investigations on α-amylase production from *G. candidum* have been performed, more specifically, using agro-industrial waste. Therefore, to fill these scientific gaps, this work aims to isolate yeasts with significant enzymatic potential for biotechnological use, to produce inexpensive α-amylase using locally available and environmentally harmful substrates, and to optimize fermentation conditions by studying their effects on the enzymatic production of the strain and improving the yield and quality of the product.

## 2. Materials and methods

### 2.1. Reagents

All bacteriological grade growth and selective media were prepared in the GMA laboratory. Dehydrated ingredients for prepared media and soluble potato starch were purchased from Panreac Química SA (Spain). Soluble starch was purchased from Biochem (France), and carboxymethyl cellulose and 3,5-Dinitrosalicylic acid were procured from Sigma-Aldrich (Germany). Other reagents and organic salts in this work were of analytical grade and commercially available (Biochem Chemopharma Co). Olive oil was obtained from a local olive oil mill (Mila, Algeria).

### 2.2. Sampling

An olive forest soil (OS) sample was collected in the region of Skikda, Algeria after removing approx. 5 cm of soil from the surface. A 95 °C thermal water (TW) sample was taken from a hot spring called Hammam El Dabbagh in the region of Guelma. Olive pomace Sigoise variety (OP), its rinse water (RW), and a mastic oil cake (MC) (*Pistacia lentiscus L*) were collected from an oil mill in the region of Mila and Skikda, respectively; these wastes were used as a source of yeast as well as a substrate for α-amylase production. Vegetable smen (VS) is one of the traditional Algerian butter products, and was purchased from supermarkets in the region of Constantine, Algeria. All samples were collected with a sterile spatula into appropriately pre-sterilized bottle containers and stored in a cool place (4 °C) until use. The pH of the samples was determined using a pH meter (GLP 21, Spain) at the laboratory level. Each sample was designated alphabetically.

### 2.3. Isolation and purification

Two methods were tested for the isolation of yeast strains. The method of successive dilutions on yeast peptone dextrose Agar (YPDA) medium [19] included spreading with a glass spreader, alongside an incubation period between three to seven days at 28 °C (Memmert INB 400, Germany). After macroscopic and microscopic observation, purification was carried out by streaking on yeast extract malt extract agar (YMA) medium and incubated until pure isolates were obtained. The pure strains were stored at 4 °C in sabouraud dextrose agar (SDA) supplemented with chloramphenicol and in yeast extract malt extract (YM) medium supplemented with 30% glycerol at −20 °C.

The enrichment method was also used for isolation. This is a procedure in which 2 mL of thermal water inoculum as the source of yeast strains was incubated in 50 mL YM broth using 250 mL Erlenmeyer flasks at 30 °C on a rotary shaker (Stuart SI500, UK) at 150 rpm for 24 hours. After incubation, the spread was performed on YM agar, YGA, and SDA, and incubated at 28 °C for three to seven days [10].

### 2.4. Screening of amylolytic strains

Starch-degrading yeasts were screened by growing in yeast extract peptone soluble starch agar (YPSA) medium containing 1% of starch as the sole carbon source [20] using the colony picking method with four quadrant isolates. The clearance zone was detected after flooding the plates with an iodine solution (1% I_2_, 2% KI), which reacts with the color of the undegraded starch.

### 2.5. Screening of cellulolytic strains

Cellulase producing yeasts were screened by growing in the yeast extract peptone carboxymethyl cellulose agar (YPCA) medium containing 1% carboxymethylcellulose (CMC) (Sigma) using the streak method with four quadrant isolates [21]. One % of Congo red solution and NaCl (1 M) for 20 minutes were used to reveal their ability to degrade cellulose. Cellulolytic activity was determined by observing a pale-yellow color around the yeast colonies.

### 2.6. Esterase activity on Tween-80 agar medium

All strains were standardized (4 McFarland) in sterile distilled water, and a total of 62 µL of the yeast suspension on 6 mm wells was inoculated into Tween-80 agar medium [21],[22]. The inoculated plates were incubated at 28 °C for 72 hours. A clear zone in the form of crystals on the Tween peptone agar (TPA) medium was observed around the colony, thus indicating either esterase or lipase production.

### 2.7. Lipase activity on olive oil agar medium

All strains were incubated on an olive oil agar medium using red phenol as a pH indicator [23],[24]. The lipolytic activity was observed by the appearance of a yellow zone. The zones of clearing, which measured <20 mm, 20–30 mm, and >30 mm, were noted as weak (+), moderate (++), and high (+++) activities, respectively.

### 2.8. Enzymatic index

The enzymatic activity index is a practical tool to improve the selection and comparison of the enzyme production of different microbial isolates. It is calculated as the relationship between the halo size and the degradation capacity of the microorganisms using the following formula:

$$Enzymatic\;index\;(EI)=\frac{Turbid\;zone\;diameter\;(mm)}{\;colony\;diameter\;(mm)}\text{ [25]}$$

### 2.9. Characterization and identification of the selected yeast strain

Yeasts with a high capacity in α-amylase production and diverse enzyme activities were identified. The identification of the selected strain was based on morphological characteristics and molecular identification. A macroscopic observation of the colonies was performed on YPD agar [26] with the naked eye, following several characteristics such as color, texture, appearance, and growth time. A microscopic observation (Leica DM1000, Germany) was performed with the 40 and 100x objectives based on the shape and mode of reproduction.

The selected yeast was subjected to molecular identification based on the amplification of the D1/D2 region of the 26S ribosomal RNA (26S rRNA) gene. DNA extraction was performed by the method of Sampaio et al. [27]. DNA amplification was performed by the PCR method using the forward primer V9G (5′-TGCGTTGATTACGTCCCTGC-3′) and the reverse primer RLR3R (5′-GGTCCGTGTTTCAAGAC-3). Sequencing of the 600–650 bp region was performed using the forward primer NL1 (5′-GCATATCAATAAGCGGAGGAAAAG-3′) and the reverse primer NL4 (5′-GGTCCGTGTTTCAAGACGG-3′; Sigma-Aldrich Co) as mentioned in the study of Turchetti et al. [28]. The PCR products were sequenced using a commercial sequencing facility (Macrogen, Amsterdam, Netherlands). The NCBI GenBank public database was used to search for sequences compatible with the obtained sequences. Then, a phylogenetic tree was constructed by the Neighbor-joining method with 1000 bootstraps [29]. The phylogenetic distances were calculated with the Kimura-2-parameter [30]. The evolutionary analyses were carried out in Molecular Evolutionary Genetics Analysis 11 (MEGA 11) (MEGA Software, Pennsylvania, USA).

### 2.10. Production of α-amylase by the selected strain

#### 2.10.1. Substrate

The fermentation studies were performed using various wastes as a basal substrate. In our study, various locally collected agricultural wastes and industrial effluents were collected from oil mills. Residues such as potato peels, leftover bread, olive pomace, and mastic oil cake were not readily available in dried form. Therefore, these collected substrates were air dried to remove the moisture content; then, grinding was performed using an electric grinder and sieved to provide a particle size ≤1 mm prior to use.

#### 2.10.2 Inoculum

For inoculum preparation, YPDA medium was used to inoculate the selected strain *Geotrichum candidum* PO27; sterile distilled water was added after incubation for 48 h at 30 °C, and the cell suspension was obtained after removing the colony from the agar using a sterile Pasteur pipette. Direct counting was performed using a Thoma cell counter. An inoculum of 10^7^ cells/mL was used [31].

#### 2.10.3 Solid state fermentation (SSF) and extraction

Solid substrates were weighed (10 g) and the desired moisture level was maintained (60%, v/w) and inoculated with 10^7^ cells/mL for an incubation period of 72 h at 30 °C. An enzyme extraction was performed by adding a 0.1 M phosphate buffer, pH 7.0, with a ratio of 1:5 (w/v). The solution was vortexed for 5 min; crude enzyme was collected from the supernatant after centrifugation (Sigma 3K15, Germany) at 10,000 rpm for 10 min at 4 °C [32]. The best substrate medium was selected for submerged fermentation.

#### 2.10.4 Submerged fermentation (SmF)

Medium of olive pomace substrate (2%–8% in distilled water) was distributed at a rate of 40 mL per 250 mL Erlen Meyer flask. After sterilization at 120 °C for 20 min and cooling, the media were inoculated (10^7^ cells/mL) and incubated at 30 °C for 72 h at 150 rpm. After fermentation, the mixture was centrifuged at 10,000 rpm, 4 °C, for 10 min and the supernatant was used to determined α-amylase activity.

#### 2.10.5 Optimization of Cultivation Conditions for α-amylase production by one factor at a time (OFAT) approach

The one factor at a time (OFAT) method was used to estimate the effects of the following conditions on a-amylase production by the *Geotrichum candidum* PO27 yeast strain: temperature, pH, nitrogen, and carbon sources. Different fermentation temperatures, from 30 °C to 65 °C, were studied to obtain the optimal incubation temperature for α-amylase. The effect of pH was studied at pH values of 4.0, 5.0, and 6.0. Buffer systems were used at a concentration of 0.1 M sodium citrate buffer to pH 4.0 and 5.0, and a sodium potassium phosphate buffer to pH 6. To determine the effects of different carbon and nitrogen sources, the basic medium was supplemented with 1% of different organic and inorganic nitrogen sources, such peptone, malt extract, yeast extract, meat extract, corn steep, KNO_3_, NaNO_3_, NH_4_Cl, and (NH_4_)_2_SO_4._ Additionally, glucose, galactose, sucrose, fructose, maltose, lactose, soluble starch, and potato starch were added at 1% to the basal fermentation media. Cultures were incubated for a period of 72 hours.

#### 2.10.6 Alpha amylase assay

The 3,5-Dinitrosalicylic acid (DNSA) method was used to quantify the produced α-amylase enzyme [33],[34]. Approximately 0.5 mL of the extract was incubated for 30 min at 40 °C with 0.5 mL of substrate (1%) prepared in (0.1 M) phosphate buffer, pH 5. The reaction was stopped by DNSA, followed by heating at 100 °C for 10 min. After cooling in an ice bath, 10 mL of distilled water was added. The absorbance was determined at 540 nm (VWR UV-1600PC Spectrophotometer, China). One unit of amylase activity is defined as the amount of amylase, which releases one µmole of maltose per min under the assay conditions. The enzymatic activity was expressed as U/gds (gram of dry substrate). All the cultures were duplicates and the results are the mean.

#### 2.10.7 Statistical analysis

The results were processed by the Minitab 19 software (Minitab, LLC, Pennsylvania, USA), which is a software designed for data analysis. All experiments were performed in duplicates, data were analyzed using an one-way ANOVA, followed by Turkey's multiple comparison test, and the statistical significance level of 0.05 was chosen. All graphics were created using GraphPad Prism 10 (Graphpad Software, LLC, Boston, USA).

## 3. Results and discussion

### 3.1. Isolation

In this study, a total of six samples were collected from several regions to obtain yeasts capable of producing α-amylase, cellulase, esterase, and lipolytic enzymes. After isolation, the pH results were taken based on the pH measurement of the samples' stock solution using a pH meter.

It was found that the four samples, namely olive pomace, rinse water, mastic oil cake, and vegetable smen, had acidic pH values of 4.40, 6.30, 6.20, and 6.33, respectively. The pH of the thermal water was neutral at 6.98, while the pH value of the olive forest soil was basic at 7.39. Generally, yeasts prefer a slightly acidic environment, and their optimal pH is between 4.5 and 5.5. Nevertheless, they grow between a wide range of pH values and adapt to pH levels between 3–10. In addition, several species can grow at strongly acidic pH values as low as 1.5‎ [35]. Generally, habitats with basic pH values are quite difficult for yeast life, while some species can grow successfully at pH levels of 10 or above [36].

**Figure 1.**
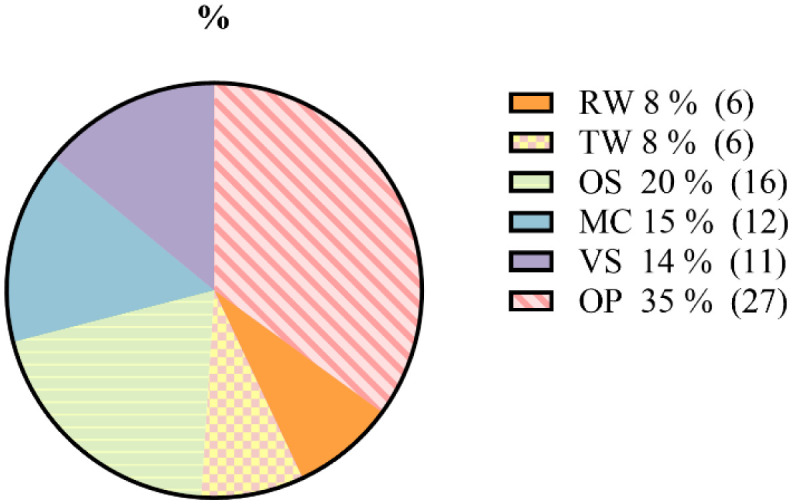
Frequency of isolated yeast strains.

From 100 isolated yeast strains, 78 yeast strains were selected for their rapid growth on YPDA (Figure 1). Based on a higher incidence, 27 yeast strains were selected from the olive pomace of isolated yeasts and six yeasts strains were isolated from olive rinse water (RW). Misbah et al's research [37] proved that RW, OP, and olive mill wastewater (OM) were favorable media for the development of enzymes producing microorganisms. Therefore, 16 strains were isolated from the soil (OS) with a similarity in colony morphology in most isolates, which agrees with the results of Williams et al. [38] who isolated 14 yeast strains obtained from palm oil impacted soil.

Twelve strains were selected from mastic oil cake (MC). To our knowledge, this source, which is used for the isolation of α-amylase, cellulase, and lipase producing yeasts, has not been previously discussed, despite their richness, as previous studies have demonstrated the importance of oily cakes as a source for the isolation of microorganisms for biotechnological use [39].

Springs hot water is one of the major sources of thermostable enzymes produced by yeasts. The isolation of yeast from TW was unsuccessful using the successive dilution technique; alternatively, an enrichment method made it possible to obtain six heat-resistant strains. Khadka et al. [10] succeeded in isolating approximately 44 bacterial strains from Kharpani hot spring water using the enrichment method, including *Geobacillus* sp. KP43, which gave a high cellulase production. Additionally, *Geobacillus* (K1C) bacteria was isolated from Manikaran hot springs and selected for its high thermostable α-amylase production [40]. Eleven strains of VS origin were obtained, and this traditional butter has been the subject of many scientific studies. However, no detailed study has ever characterized its microbial diversity, including yeasts. In the studies cited above, the microbial diversity mainly focused on the diversity of proteolytic lactic acid bacteria and, in a few cases, yeasts in traditional Algerian butter and on yeast diversity in different types of cheeses [41],[42].

### 3.2. Screening of enzymes producing yeasts

**Figure 2.**
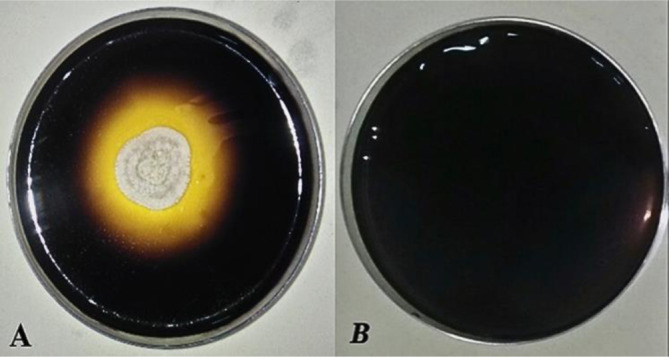
Amylase qualitative assay for the selected isolate PO27 (A: Presence of selected yeast, B: Absence of selected yeast‎).

Hydrolysis starch by α-amylase produced a light purple area around the colony after revelation with the Lugol solution. The absence of a clear zone indicated a reaction between iodine reagents and non-hydrolyzed starches in the Starch Agar medium [43]. The iodine-starch reaction is due to the formation of helical amylose and iodine as I_3_ that fills the helical nucleus [44]. Halo zone formation and amylolytic index (AI) were used as a semi-quantitative method to classify isolates as being highly amylase producers (AI > 1.5) [45]. Twenty-seven yeast strains were able to hydrolyze starch; among these strains, RW1-3, RW1-2, and PO 27, showed high amylolytic activity (Figure 2).

**Figure 3.**
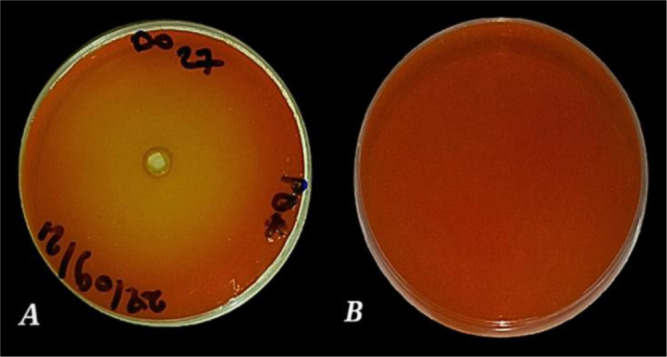
Lipase qualitative assay for the selected isolate PO27 (A: Presence of ‎olive oil, B: Absence of olive oil).

**Figure 4.**
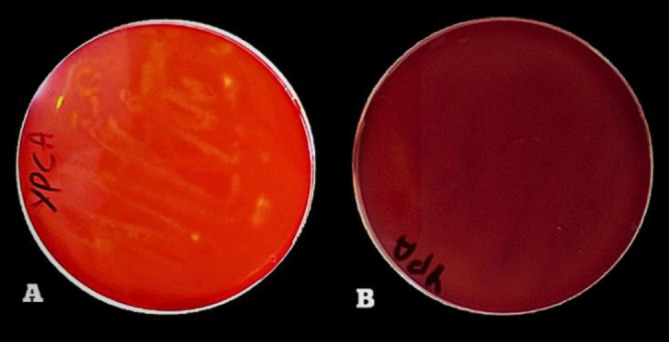
Cellulase qualitative assay for the selected isolate PO27 (A: Presence of ‎carboxymethyl cellulose‎, B: Absence of carboxymethyl cellulose).

**Figure 5.**
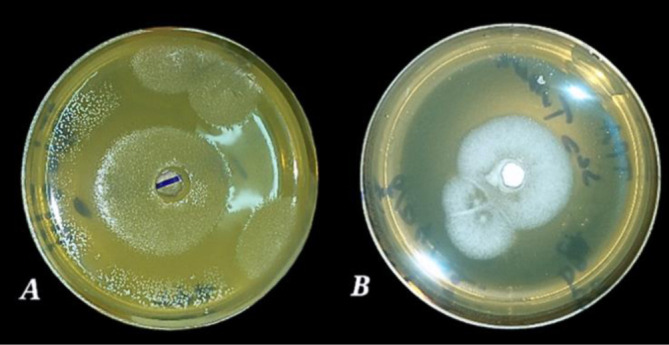
Esterase qualitative assay for the selected isolate PO27 (A: Presence of ‎Tween 80, B: Absence of Tween 80).

**Figure 6.**
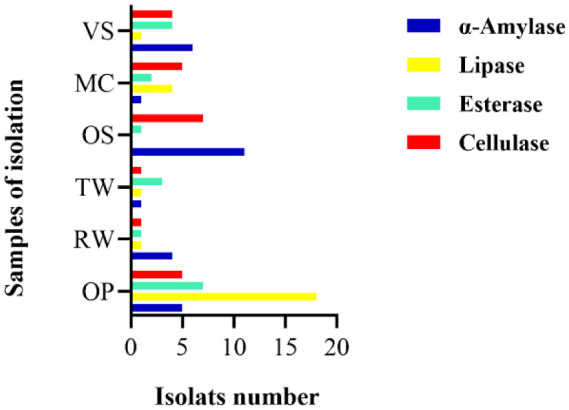
Distribution of enzymatic activities on the six samples. ‎Bars: amylolytic isolated strains (blue bar), ‎lipolytic isolated strains (yellow bar), esterolytic isolated strains (green bar), cellulolytic isolated strains (red bar).

Olive oil mixed with agar medium represents a good choice for selecting lipase-positive strains [46] with an indicator such as red phenol [47]. After incubation on an olive oil medium, the dishes displayed yellow areas around the wells due to free fatty acids released by the lipolytic organisms, which lowered the pH of the medium from phenol red to yellow, thus indicating the presence of lipase [46]. Twenty-five strains demonstrated lipolytic activity. However, the PO27 strain was the most efficient because it showed the highest diameter on olive oil (Figure 3). All other strains exhibited low and moderate lipolytic activity. The clear zone around the colony showed that the isolates were also capable of promoting cellulase hydrolysis [48]. Among the 78 yeast strains, most had no cellulase activity except five strains, namelyPO27, PO6, PO8, PO20, and RW2-2, which all showed weak activities (Figure 4). Additionally, Tween-80 was used as a substrate for esterase or lipase screening. No color is required for visualization; they provide the opaque zones around the colonies, which indicate the precipitation zones of the calcium salt, while hydrolysis indicates either esterase or lipase activity [49] (Figure 5). Seventeen strains showed esterase activity, and all species exhibited moderate and high esterase activity, including the amylolytic, cellulolytic, and lipolytic yeast isolate PO27 (Figure 6).

In addition, yeast biodiversity varied between the six isolation samples (Figure 6). The results of the experiment revealed that most of them present a good biotechnological interest due to their capacity to tolerate high yeast concentrations. However, the maximum number of yeast colonies obtained from the olive pomace sample indicates that the majority of them have a more diversified and important enzymatic potential.

As a result, the olive pomace origin of the yeast PO27 strain was selected for its high capacity to produce α-amylase and its enzymatic diversity profile. The PO27 isolate showed a 40 mm α-amylase halo and a 1.6 of AI, as well as a 38 mm lipase halo and 22 mm opaque halo in the Tween substrate with 1.1 of enzymatic index. Moreover, it showed a positive result with the cellulase qualitative test, and which is not interesting for industrial use because of its low activity. Following the screening and identification of the isolate, it was found that our results are consistent with those of previous studies [50], in which isolated *Geotrichum candidum* showed an ability to hydrolyze and assimilate several carbon sources including starch, Tween, casein, and CMC through their production of enzymes such as amylase, lipase (or esterase), protease, and cellulase. Furthermore, there are many reports in the literature about the production of lipases from *Geotrichum candidum* [51]. Different strains have shown the enzymatic potential of this yeast species, including *Geotrichum candidum* CMSS06, which produced α-amylase [52], *Geotrichum candidum* Strain Gad1, which is able to produce cellulase [53], *Geotrichum candidum* 3C, which produced endo-1,4-xylanase [54], *Geotrichum candidum* AA15, which also exhibited the capacity to produce pectinase [55], and *Geotrichum candidum* QAUGC01, which allowed for the production of serine alkaline protease [56].

### 3.3. Identification and characterization of the selected yeast strain

**Figure 7.**
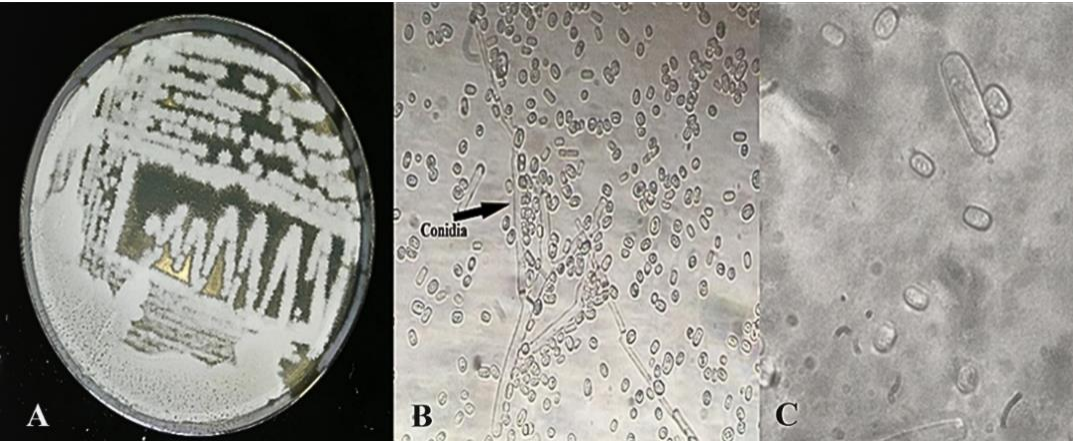
Morphology observation of PO27 strain cultured on YPD agar plate for 48 h at 30 °C (A: Macroscopic observation, B: ‎Microscopic observation G:40X, C: Microscopic observation G:100X).

The species presents a yeast with a fuzzy, filamentous appearance, with a mold-like aspect, in addition to its rapid growth; the colonies are white and have a velvety white cottony mycelium, as described by Attanayaka et al. [52]. Under the light microscope, its shape is rectangular and then rounded, and showed many arthrospores, which are consistent with a previous study [57] that clearly showed conidia (arthrospores) with variable width. Based on morphological characterization, this yeast species was identified as *Geotrichum* sp. (Figure 7). The species has been confirmed by molecular identification.

**Figure 8.**
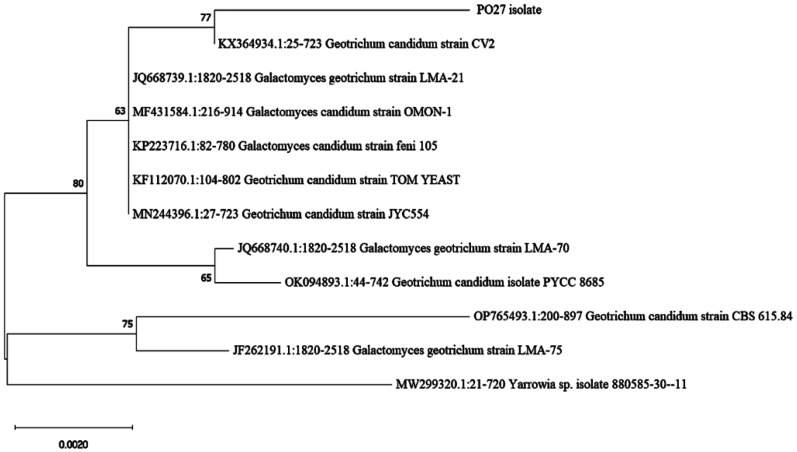
Phylogenetic relationships of PO27 and other closely related *Geotrichum* sp. using NJ ‎method‎ with ‎bootstrap value of 1000 ‎replicates. *Yarrowia* sp. was used as outgroup. ‎Bootstrap values (>50%) were ‎‎shown at the nodes. Scale bar, 0.002 nucleotide substitution ‎rate units.

After molecular identification from sequencing of the D1/D2 domain of the 26S gene sequence, the length of the D1/D2 region was found to be 699 bp (is included in supplementary D1/D2 region‎). Sequence comparison of the PO27 isolate (GenBank accession no. PP024529) with those included in the GenBank database ‎showed a 99.57% similarity with the *Geotrichum candidum* strain CV2 (GenBank Accession no. KX364934) and illustrated a high similarity to its teleomorph *Galactomyces candidum*. According to the phylogenetic analysis (Figure 8), the isolate PO27 clustered in a branch near to *G. candidum* with more than 77% probability, which is well supported. Based on its morphological and molecular characteristics, we identified the isolate PO27 as *Geotrichum candidum* and not as its teleomorph *Galactomyces candidum*, since no ascospores were observed.

### 3.4. Production of α-amylase studies

**Figure 9.**
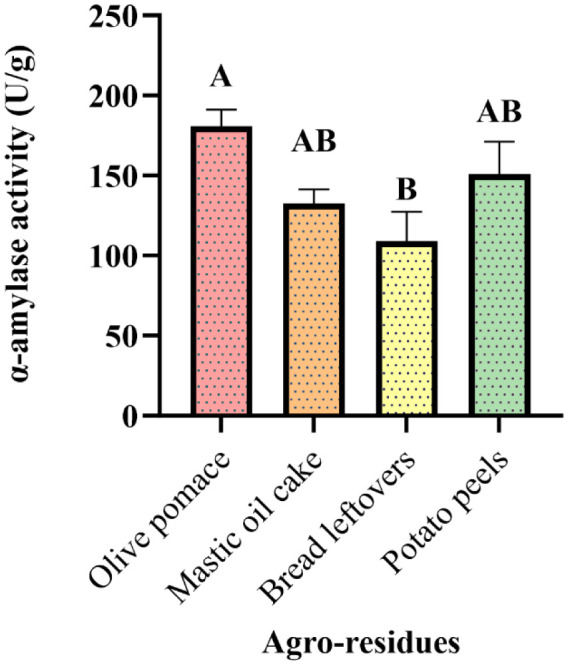
α-amylase production by *G. candidum* PO27 under solid-state ‎fermentation (Tukey method: Means that do not share a letter are significantly ‎different).

In SSF, the most important factor is the choice of a suitable medium for the production of an enzyme. In this study, the selected strain *G. candidum* PO27 was inoculated into solid residues such as potato peels, bread leftovers, olive pomace, and mastic oil cake. Then, the produced α-amylase was quantified via the DNSA method. Among the four agro-substrates tested, a significant difference (p = 0.038) was obtained with olive pomace 180.71 U/g to be the best solid substrate for the α-amylase production, followed by potato peels 150.63 U/g, mastic oil cake 132.66 U/g, and bread leftovers 109.04 U/g (Figure 9). This can be explained by the differences in the texture and chemical composition of the substrates.

The amylases production from agro-industrial waste is intended to resolve pollution problems and to obtain a low-cost medium. Literature reports suggest that *Aspergillus awamori* isolated from olive cake can produce the highest amylase activity of 230 U/g [58]. In contrast to Obi et al. [59], the potato peel was suggested as the best substrate with the highest amylase production (2.36 U/mL) from *Bacillus subtilis*. Using various oil cakes as low-cost substrates, such as groundnut oil cake (GOC), coconut oil cake (COC), and sesame oil cake (SOC) *by Aspergillus oryzae*, Balakrishnan et al. [60] also studied α-amylase production under SSF. Their results showed that oil cake (GOC) was the best substrate for a maximal α-amylase production of 9868.12 U/g, followed by 4031.12 U/g using COC and 3068.15 U/g using SOC.

In another study, using *Bacillus subtilis* [61] to produce α-amylase, it was shown that stale bread had the highest amylolytic activity (107.3 U/min), whereas the potato peel yielded 55.5 U/min. Furthermore, Benabda et al. [62] demonstrated that SSF from *Rhizopus oryzae* produced α-amylase (100 U/g) using bread waste as a substrate, which is very similar to our SSF results on leftover bread. Agricultural substrates such as wheat bran, rice bran, maize bran, corn bran, and wheat straw have attracted attention for amylase production [63]. Moreover, the capacity of several fruit peels, including banana, orange, and pineapple peels, to provide alternative carbon sources for α-amylase production was examined [64],[65]. Additionally, Singh et al. [66] found that SSF of an apple peel could yield 17468 U/L of α-amylase from the *Bacillus subtilis* BS1934 strain. In another study, watermelon rinds (WMR) were used to produce α-amylase from *Trichoderma virens* [67].

**Figure 10.**
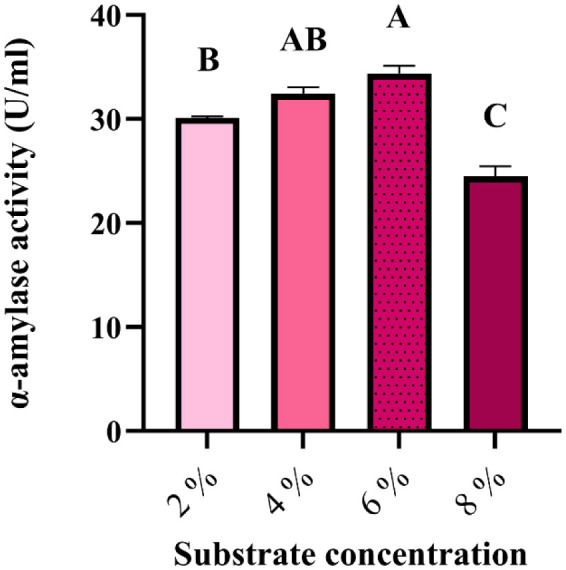
α-amylase production by *G. candidum* PO27 under submerged ‎fermentation (Tukey method: Means that do not share a letter are significantly ‎different).

In order to choose the best conditions for enzymatic production, submerged fermentation was performed at different olive pomace substrate concentrations from 2% to 8%. The analysis of the experimental results by the ANOVA reveals that the difference in activity as a function of substrate concentration is highly significant (p = 0.000), where a 6% concentration was the best with 34.395 U/mL, compared to other concentrations, followed by 4% with 32.8 U/mL. In comparison, 2% and 8% exhibited low activities with 30.115 U/mL and 24.490 U/mL, respectively (Figure 10).

Comparing our results is challenging because this is the first study to use *G. candidum* as a source of α-amylase by submerged fermentation using olive pomace. In addition, it is necessary to consider that our study is very attractive because of the dispensation of any mineral supplementation that makes it a suitable low-cost environment for α-amylase production by the *G. candidum* PO27 strain (34.395 U/mL). Using a synthetic media, the highest production of the species *Geotrichum candidum* CMSS06 reached 6.4 U/mL of α-amylase at 72 h, whereas *Aspergillus* spp. showed a maximum activity of 1.2335 U/mL at 96 h‎ [52]. Compared to Divya and Padma [68], which used a synthetic YEPD broth supplemented with starch, the amylase activity was 130 U/mL by the isolate *Geotrichum* sp. In contrast to Falih [69], soil yeast *Geotrichum candidum* was found to have an amylase activity of 50 µg/mL using the Czapek-Dox medium, while the maximum amylolytic activity of *Geotrichum capitatum* was 34 µg/mL. Moreover, the highest growth rate of *Saccharomyces cerevisiae* has been observed in the treatment with 2% potato peels in SmF, which increased amylase activity [70]. Because of their low cost, availability, and simplicity, oil cakes such as cocos nut oil cake were used as a carbon source in liquid fermentation to produce α-amylase by *Aspergillus flavus*, which showed a higher enzyme activity (170.3 µg/mL) [71].

**Figure 11.**
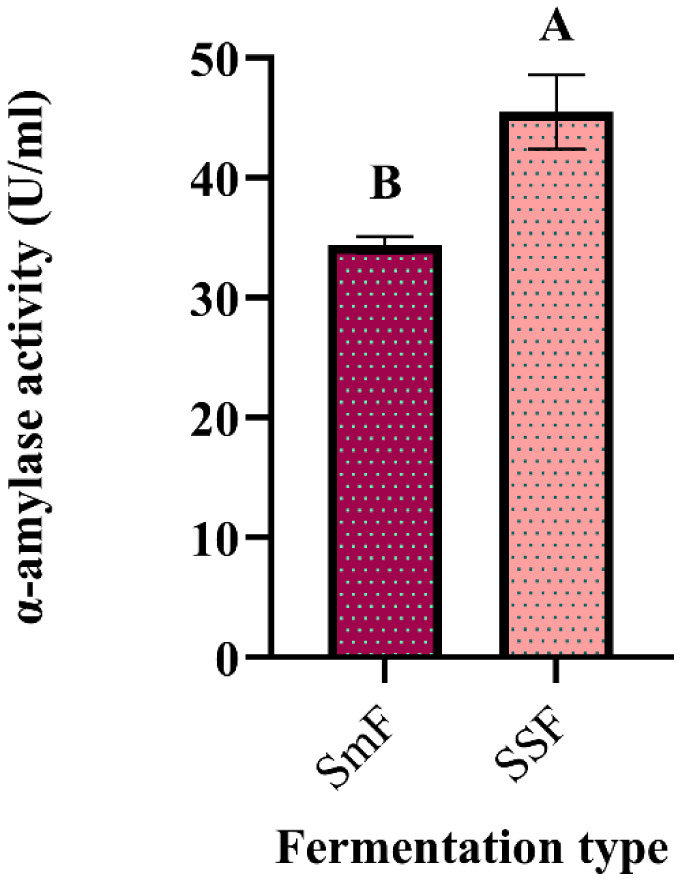
Comparison of α-amylase production by *G. candidum* PO27 in solid state and ‎submerged fermentation (Tukey method: Means that do not share a letter are ‎significantly different).

In recent years, the technique of the SSF process has been developed and used more extensively because of its simplicity, low cost, the simple need for fermentation equipment, improved productivity, and decreased water production [18]. As shown in Figure 11, the α-amylase activity in SSF (45.47 U/mL) was highly significant (p = 0.038) when compared with the activity in SmF (34.395 U/mL). This result corroborates that of Jesubunmi and Ogbonna [72], who reported that the production of glucoamylase and cellulase by both *Fusarium* sp. and *Rhizopus* sp. was significantly higher in solid state culture (p < 0.05) than in suspended culture. However, that did not prevent about 90% of industrially important enzymes to have traditionally been produced by SmF because of ease of handling and sterilization and a better control of environmental factors such as temperature and pH [34]. Furthermore, Premalatha et al. [73] reported that the production of extracellular α-amylase from *Aspergillus tamarii* using wheat bran (WB) performed better in the SSF method than with SmF and achieved a higher α-amylase activity (519.40 U/g).

### 3.5. Optimization of Cultivation Conditions for α-amylase production by one factor at a time ‎‎‎(OFAT) approach‎

#### 3.5.1. Temperature effect on α-amylase production

**Figure 12.**
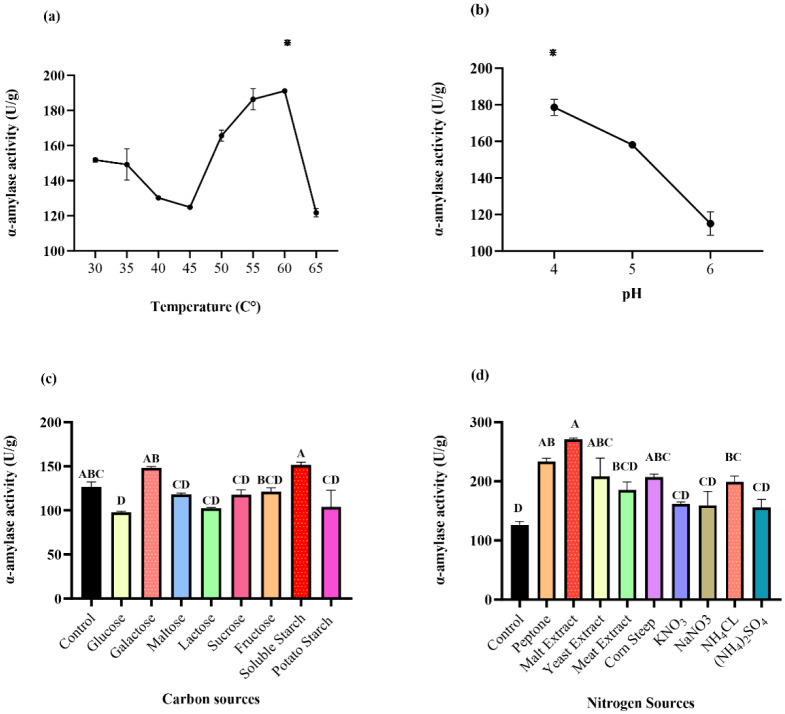
Optimization of α-amylase production by *Geotrichum candidum* PO27 by OFAT approach. Effect of (a) Temperature, (b) pH, (c) Carbon sources, (d) Nitrogen sources on α-amylase production, (*) indicate significant difference, (Tukey method: Means that do not share a letter are ‎significantly different ‎p < 0.05).

The amylolytic strain seems to be thermophilic. The maximal activity was obtained (191.10 U/g) at 60 °C (p = 0.000), followed by 186.35 U/g at 55 °C (Figure 12a). Enzyme production dropped at a temperature of 65 °C and gave an activity level of 121.68 U/g, thus indicating the inhibition of amylase production, probably by suppressing cell viability and enzyme inactivation [74]. These results agree with several studies. Luang-In et al. [75] ‎showed that the optimal temperature for amylase of *Bacillus* sp. 3.5AL2 was 60 °C; moreover, Finore et al. [76] ‎obtained maximal α-amylase secretion from *Anoxybacillusamylolyticus* at 60 °C. The results showed that *G. candidum* PO27 α-amylase is a thermophilic enzyme with potential use in industrial processes. Microorganisms able to grow optimally at temperatures between 50 °C and 60 °C are known as moderate thermophiles. It can be assumed that moderate thermophiles, which are closely related phylogenetically to mesophilic organisms, can adapt to life in warm environments [77].

#### 3.5.2. pH effect on α-amylase production

An optimal pH is an essential factor for the stability of the enzyme produced. Enzymes are pH sensitive, and therefore production process pH must be carefully controlled‎ [78]‎. The extracellular α-amylase activity was significantly higher (p = 0.002) at a pH 4 (178,61 U/g), followed by a pH of 5 (158,145 U/g); however, the enzymatic activity was considerably lower at a pH of 6 (115,075 U/g), which indicates the acidophilic nature of the isolated *G. candidum* PO27 (Figure 12b). These results are similar to Tatsinkou Fossi‎ et al‎. [79], who found that a pH 4.5 was optimal for amylase production at 30 °C by an isolated yeast. Additionally, Olakusehin and Oyedeji ‎[80] ‎revealed that the optimal pH for α-amylase production by *Aspergillus flavus* S2-OY was found to be at a pH of 5. On top of that, the pH measurement of the optimized medium of the *G. candidum* CMSS06 strain proved that the strain appropriates the acidic medium as the pH of the culture supernatant dropped from 4.0 to 3.46 [52].

#### 3.5.3. Carbon source effect on α-amylase production

The carbon source is one of the major factors affecting enzyme production, particularly when it performs an inducer role. The α-amylase production by the G. *candidum* PO27 strain was significantly (p = 0.000) higher in the presence of soluble starch (151, 54 U/g), followed by galactose (148,315 U/g); however, the enzymatic activity was considerably lower in addition to the other carbon source as compared to the SSF without carbon supplementation (Figure 12c). This can be explained by the high carbohydrate content of olive pomace [81]‎, which is effective for the α-amylase production. This enzyme is extracellular, and its production is induced by its substrate at a certain limit concentration [65]. In addition to its role as an inducer, starch stabilizes the enzyme‎ [82]. This result is similar to Almanaa et al.‎ ‎[64], ‎who produced maximal amounts of amylase from *Bacillus subtilus* D19 using starch as the carbon source. Moreover, the α-amylase production from *Streptomyces* sp. Al-Dhabi-46 was found to be maximal in the culture medium containing 1% starch as carbon source (208 ± 11.4 U/mL) [83]‎. In addition, galactose had a positive impact as the highest enzymatic performance (944 U/gds) by *Penicillium chrysogenum* in SSF **‎**[84].

#### 3.5.4. Nitrogen source effect on α-amylase production

Due to the low nitrogen content of olive pomace, the α-amylase ‎production was enhanced by the addition of various nitrogenous compounds. From the result of the Figure 12, it appears that *G. candidum* PO27 can use malt extract to significantly (p = 0.000) enhance α-amylase production and achieve maximal production (270,52 U/g). With few investigating the use of malt extract as a nitrogen source to improve amylase production, maximal α-amylase production using malt extract by *Pseudomonas balearica* VITPS19 has been reported by Kizhakedathil and Subathra Devi‎ [85]‎. Although it served as the inorganic source, NH_4_Cl gave the best α-amylase production (199.18 U/g) compared with the other inorganic sources used. Ahmed et al. ‎[86] revealed its insignificant effect on bacterial α-amylase production.

## 4. Conclusions

Several yeast strains were isolated from olive pomace, olive forest soil, thermal water, olive rinse water, ‎mastic oil cake, and vegetable smen. They were all capable of producing α-amylase, esterase, lipase, ‎and cellulase. Among these isolates, the PO27 strain showed higher amylolytic activity and presented ‎an important enzymatic diversity. This isolate was identified as *G. candidum* ‎according to morphological characteristics and was confirmed by 26S rRNA gene sequencing. ‎The present study clearly indicates that *G. candidum* PO27 isolated from olive pomace showed a ‎significant capacity in the production of α-amylase by degrading various agro-industrial wastes ‎as natural substrates. Among the cheap sources tested, olive pomace was best for α-amylase ‎production, and the best activity of α-amylase was obtained by SSF compared ‎with SmF. At the same time, the effects of physicochemical factors on PO27 α-amylase production were studied. The α-amylase production process by *G. candidum* ‎PO27 using this waste can be optimized using a statistical design, purified, tested, and scaled up for industrial ‎production, thus bringing environmental benefits to energy security. ‎

## Use of AI tools declaration

The authors declare they have not used Artificial Intelligence (AI) tools in the creation of this article.


