# Supplementary material for: Extracellular enzymes producing yeasts study: cost-effective production of α-amylase by a newly isolated thermophilic yeast Geotrichum candidum PO27
Source: AIMS Microbiol. 2024 Jan 29;10(1):83–106. doi: 10.3934/microbiol.2024006 (PMC10955176; doi:10.3934/microbiol.2024006)
Supplement: Supplementary file 1 [file microbiol-10-01-006-s001.pdf]

AIMS Microbiology

AIMS Microbiology, 10(1): 83–106.
DOI: 10.3934/microbiol.2024006
Received: 22 November 2023
Revised: 01 January 2024
Accepted: 22 January 2024
Published: 29 January 2024

http://www.aimspress.com/journal/microbiology

Research article

Extracellular enzymes producing yeasts study: cost-effective production of α-amylase by a newly isolated thermophilic yeast *Geotrichum candidum* PO27

Ibtissem Chaib[1,*], Scheherazed Dakhmouche-Djekrif [1,2], Leila Bennamoun [1] and Tahar Nouadri[1]

[1] Laboratory of Microbiological Engineering and Applications, Department of Biochemistry and Molecular and Cellular Biology, Faculty of Natural and Life Sciences, Frères Mentouri University Constantine 1, Constantine 25017, Algeria
[2] Department of Natural Sciences, Teachers Training School El Katiba Assia Djebar, University town Ali Mendjeli, Constantine 25000, Algeria

* **Correspondence:** ibtissem.chaib@doc.umc.edu.dz; Tel: +21354166011.

D1/D2 region

>Geotrichum_candidum_PO27
AAAATAATCAAAACTTTTAACAATGGATCTCTTGGTTCTCGTATCGATGAAGAACGCAGC
GAAACGCGATATTTCTTGTGAATTGCAAAAGTGAATCATCAGTTTTTGAACGCACATTGC
ACTTTGGGGTATCCCCCAAAGTATACTTGTTTGAGCGTTGTTTCTCTCTTGGAATTGCATT
GCTTTTCTAAAATTTCGAATCAAATTCGTTTGAAAAACAACACTATTCAACCTCAAATCAA
GTAGGATTACCCGCTGAACTTAAGCATATCAATAAGCGGAGGAAAAGAAACCAACAGGG
ATTGCCTTAGTAACGGCGAGTGAAGCGGCAAAAGCTCAAATTTGAAATCGGCCACCAGG
TCGAGTTGTAATTTGTAGATGGTATCTGGAGAGCGGATTAAAGTCTGTTGGAACACAGCG
CCTTAGAGGGTGACAGCCCCGTAAAATCTATTCTCATTGTAAGATACTTTCGAAGAGTCGA
GTTGTTTGGGAATGCAGCTCTAAGTGGGAGGTAAATTCCTTCTAAAGCTAAATATTGACG
AGAGACCGATAGCGAACAAGTACTGTGAAGGAAAGATGAAAAGCACTTTGAAAAGAGA
GTGAAAAAGTACGTGAAATTGTTAAAAGGGAAGGGTATTGAATCAGACTTGGTGCTGTT
GTTCAACTGTGTTTCGGCATAGTGTACTCAGCAGTACTAGG.

AIMS Press

© 2024 the Author(s), licensee AIMS Press. This is an open access article distributed under the terms of the Creative Commons Attribution License (http://creativecommons.org/licenses/by/4.0)
